# Supplementary material for: Comprehensive Metabolite Profiling in Genetic Resources of Garlic (Allium sativum L.) Collected from Different Geographical Regions
Source: Molecules. 2021 Mar 5;26(5):1415. doi: 10.3390/molecules26051415 (PMC7962061; doi:10.3390/molecules26051415)
Supplement: Supplementary file 1 [file molecules-26-01415-s001.pdf]

# Comprehensive Metabolite Profiling in Genetic Resources of Garlic (*Allium sativum* L.) Collected from Different Geographical Regions

**Mostafa Abdelrahman [1,†], Sho Hirata [2,†], Takuya Mukae [3], Tomohiro Yamada [4], Yuji Sawada [5], Magdi El-Syaed [6] Yutaka Yamada [5], Muneo Sato [5], Masami Yokota Hirai [5] and Masayoshi Shigyo [3,*]**

[1] Botany Department, Faculty of Science, Aswan University, Aswan 81528, Egypt; meettoo2000@yahoo.com
[2] Laboratory of Agroecology, Faculty of Agriculture, Kyushu University, Kasuya, Fukuoka 811-2307, Japan; hirata.sho.481@m.kyushu-u.ac.jp
[3] Laboratory of Vegetable Crop Science, Graduate School of Sciences and Technology for Innovation, College of Agriculture, Yamaguchi University Yamaguchi City, Yamaguchi 753-8515, Japan; a003wfu@yamaguchi-u.ac.jp
[4] Allium Unit, Division of Vegetable Breeding, Institute of Vegetable and Floriculture Science, National Agriculture and Food Research Organization (NARO), 360 Kusawa, Ano, Tsu, Mie 514-2392, Japan; yamatomo@affrc.go.jp
[5] RIKEN Center for Sustainable Resource Science, 1-7-22 Suehiro-cho, Tsurumi-ku, Yokohama, Kanagawa 230-0045, Japan; yuji.sawada@riken.jp (Y.S.); yutaka.yamada@riken.jp (Y.Y.); muneo.sato@riken.jp (M.S.); masami.hirai@riken.jp (M.Y.H.)
[6] Molecular Biotechnology Program, Field of Advanced Basic Sciences, Galala University, New Galala City, Egypt; magdiel_sayed@gu.edu.eg
* Correspondence: shigyo@yamaguchi-u.ac.jp; Tel.: +81-839-335-842
† Equally contributed.

**Supplementary Table S1.** Total saponin and fructan contents in the investigated garlic accessions.

| World Collection No. | Sample name | Saponin (mg・$g^{-1}$ DW)* | Fructan (g・$100g^{-1}$ FW) |
|---|---|---|---|
| AsWC146 | JPN37 | 18.00 | 18.52 |
| AsWC147 | JPN39 | 3.36 | 18.12 |
| AsWC148 | JPN40 | 12.81 | 19.48 |
| AsWC149 | TWN45 | 13.49 | 18.22 |
| AsWC150 | CHN54 | 8.05 | 17.62 |
| AsWC151 | EGY55 | 4.27 | 16.41 |
| AsWC154 | JPN65 | 7.82 | 18.91 |
| AsWC158 | PER137 | 9.48 | 14.47 |
| AsWC159 | CHN180 | 10.67 | 18.69 |
| AsWC162 | GRE307 | 17.55 | 18.36 |
| AsWC169 | EGY489 | 32.18 | 12.16 |
| AsWC173 | CHN524 | - | 19.18** |
| AsWC175 | TUR542 | 3.38 | 14.91** |
| AsWC177 | GER556 | 9.83 | 10.93 |
| AsWC181 | F115 | 18.21 | 14.19 |
| AsWC183 | F138 | 17.21 | 13.97 |
| AsWC186 | F189 | 6.86 | 2.58 |
| AsWC189 | F1-200-34 | 17.53 | 10.47 |
| AsWC193 | Fs407 | 9.60 | 23.00 |
| AsWC195 | Fs414 | 10.10 | 10.24 |
| AsWC196 | Fs422 | 12.73 | 11.33 |
| AsWC198 | SAN BARTOROME（Gatur） | 23.68 | 15.96 |
| AsWC201 | SYR_No.5 | 29.82 | - |
| AsWC202 | IND-IIT | 7.12 | 10.36 |
| AsWC205 | VNM_Mai Dinh | 17.71 | 10.47 |
| AsWC208 | THA67-4 | - | - |
| AsWC209 | THA16-5 | - | 11.08 |
| AsWC214 | JPN_Taishu-san | 30.41 | 11.97 |
| AsWC224 | SHA_Shanghi | - | 7.30 |
| AsWC226 | JPN_Kikai-onodu | 24.96 | - |
| | Mean<br>Standaed Deviation<br>Standard Error | 14.49<br>8.29<br>1.63 | 14.19<br>4.63<br>0.93 |

-: No data.

*The data of saponin contents were derived from Table 5 of the doctoral thesis (51). **The fructan contents of the growth results at Yamaguchi in 2011 for two accessions, namely CHN524 and TUR542 were obtained from Figure 17 of the doctrol thesis (51).

**Supplementary Table S2.** List of normalized signal intensities of the metabolites identified in 30 garlic accessions

| ID | Annotation | JPN37 | JPN39 | JPN40 | TWN45 | CHN54 | EGY55 | JPN65 | PER137 |
|---|---|---|---|---|---|---|---|---|---|
| X00004 | 1-Amino-1-cyclopentanecarboxylic acid | 5.11 | 4.51 | 9.61 | 6.02 | 8.38 | 7.18 | 4.55 | 5.89 |
| X00010 | Citric acid,Anhydrous | 2.57 | 2.30 | 1.33 | 2.27 | 4.24 | 2.94 | 3.25 | 0.71 |
| X00039 | Adenine | 0.69 | 0.59 | 0.54 | 0.85 | 0.73 | 1.18 | 1.21 | 0.54 |
| X00049 | Glycine | 0.15 | 0.09 | 0.32 | 0.20 | 0.19 | 0.36 | 0.24 | 0.19 |
| X00054 | L-(-)-Phenylalanine | 1.17 | 1.14 | 1.20 | 1.02 | 0.98 | 2.27 | 3.40 | 2.50 |
| X00055 | L-Tryptophane | 2.94 | 0.64 | 0.79 | 1.38 | 2.39 | 0.89 | 2.20 | 0.63 |
| X00056 | L-Tyrosine | 0.31 | 0.22 | 0.32 | 0.49 | 0.65 | 0.40 | 0.94 | 0.30 |
| X00060 | Sucrose | 7.57 | 5.77 | 5.00 | 5.39 | 4.60 | 6.63 | 5.31 | 5.37 |
| X00095 | Methylmalonic acid | 0.42 | 0.49 | 0.34 | 0.40 | 0.40 | 0.44 | 0.33 | 0.35 |
| X00099 | Pyridoxamine dihydrochloride | 0.04 | 0.05 | 0.10 | 0.05 | 0.06 | 0.10 | 0.08 | 0.07 |
| X00187 | Cystathionine | 0.02 | 0.01 | 0.02 | 0.01 | 0.01 | 0.02 | 0.02 | 0.02 |
| X00188 | Cytidine,cell culture tested | 1.42 | 0.92 | 1.36 | 1.32 | 1.42 | 2.99 | 4.01 | 0.76 |
| X00212 | DL-2,3-Diaminopropionic acid monohydrochloride | 0.17 | 0.14 | 0.11 | 0.11 | 0.20 | 0.14 | 0.11 | 0.04 |
| X00218 | DL-threo-beta-Methylaspartic acid | 0.12 | 0.09 | 0.07 | 0.10 | 0.10 | 0.10 | 0.05 | 0.06 |
| X00241 | Guanosine | 0.20 | 0.33 | 0.25 | 0.27 | 0.17 | 0.43 | 0.20 | 0.20 |
| X00256 | Inosine | 0.10 | 0.25 | 0.12 | 0.13 | 0.07 | 0.24 | 0.08 | 0.06 |
| X00267 | L-Asparagine | 2.55 | 1.46 | 3.99 | 1.93 | 4.13 | 3.06 | 3.14 | 4.70 |
| X00268 | L-Aspartic acid | 5.93 | 4.91 | 5.23 | 4.67 | 7.65 | 3.31 | 3.79 | 4.80 |
| X00270 | L-Cystine | 0.05 | 0.07 | 0.04 | 0.10 | 0.04 | 0.10 | 0.05 | 0.06 |
| X00272 | L-Glutamic acid | 39.38 | 38.09 | 47.04 | 50.36 | 56.08 | 54.88 | 37.35 | 35.72 |
| X00273 | L-Glutamine | 22.26 | 17.12 | 51.20 | 20.34 | 33.05 | 33.04 | 23.88 | 32.13 |
| X00285 | L-Ornithine monohydrochloride | 0.53 | 0.26 | 0.75 | 0.44 | 0.98 | 0.73 | 0.70 | 0.98 |
| X00286 | L-Proline | 14.70 | 5.61 | 17.01 | 9.37 | 7.17 | 17.40 | 18.81 | 9.62 |
| X00287 | L-Pyroglutamic acid | 5.55 | 4.93 | 10.94 | 6.92 | 10.31 | 7.73 | 4.59 | 6.58 |
| X00288 | L-Serine | 6.95 | 4.56 | 9.66 | 4.76 | 6.39 | 10.41 | 9.83 | 7.57 |
| X00338 | S-Carboxymethyl-L-cysteine | 0.18 | 0.14 | 0.19 | 0.10 | 0.19 | 0.12 | 0.17 | 0.15 |
| X00344 | Succinic acid | 0.39 | 0.49 | 0.34 | 0.40 | 0.46 | 0.42 | 0.33 | 0.35 |
| X00364 | Tyramine | 0.52 | 0.41 | 0.47 | 0.41 | 0.76 | 1.58 | 0.88 | 0.85 |
| X00372 | Uridine-5'-monophosphate | 0.04 | 0.03 | 0.03 | 0.02 | 0.06 | 0.03 | 0.03 | 0.03 |
| X00379 | alpha-Lactose monohydrate | 0.64 | 0.56 | 0.47 | 0.50 | 0.43 | 0.65 | 0.46 | 0.48 |
| X00389 | gamma-Amino-n-butyric acid | 4.07 | 3.21 | 2.27 | 2.56 | 4.83 | 3.26 | 2.45 | 1.32 |
| X00392 | 6-Aminohexanoic acid | 0.72 | 0.36 | 0.48 | 0.64 | 0.96 | 0.58 | 1.40 | 0.47 |
| X00416 | Keracyanin Chloride | 4.82 | 2.17 | 3.36 | 2.32 | 1.87 | 4.82 | 0.75 | 2.33 |
| X00419 | Isorhamnetin-3-O-glucoside | 3.00 | 1.04 | 1.06 | 4.42 | 1.55 | 2.31 | 7.34 | 2.19 |
| X00422 | Kaempferol-3-O-glucoside | 10.16 | 5.34 | 12.19 | 11.82 | 10.26 | 14.63 | 14.67 | 9.57 |
| X00432 | Hyperoside | 1.69 | 0.56 | 0.80 | 2.08 | 0.65 | 1.70 | 5.25 | 1.39 |
| X00477 | O-Acetyl-L-serine hydrochloride | 0.51 | 0.47 | 0.42 | 0.44 | 0.43 | 0.63 | 0.44 | 0.48 |
| X00502 | L-Saccharopine | 0.19 | 0.16 | 0.23 | 0.31 | 0.39 | 0.26 | 0.42 | 0.18 |
| X00675 | 5'-Deoxy-5'-Methylthioadenosine | 2.42 | 1.36 | 2.38 | 1.56 | 2.06 | 2.60 | 2.16 | 1.45 |
| X00695 | L-allo-threonine | 2.61 | 1.82 | 2.62 | 2.35 | 3.11 | 2.61 | 2.68 | 2.15 |
| X00755 | 5-Aminovaleric acid | 2.04 | 1.80 | 2.39 | 2.27 | 3.94 | 2.44 | 3.57 | 2.28 |

| | | | | | | | | | |
|---|---|---|---|---|---|---|---|---|---|
| X00797 | L-Threonic acid hemicalciumsalt | 0.86 | 0.77 | 0.48 | 0.84 | 0.81 | 0.92 | 0.57 | 0.46 |
| X00849 | Hesperidin | 0.35 | 0.08 | 0.07 | 0.34 | 0.04 | 0.26 | 0.10 | 0.27 |
| X00853 | Luteolin-3',7-di-O-glucoside | 4.86 | 3.43 | 6.31 | 6.02 | 4.79 | 7.41 | 8.45 | 4.27 |
| X00854 | Luteolin-4'-O-glucoside | 21.85 | 11.97 | 28.01 | 26.62 | 22.11 | 33.31 | 34.37 | 21.09 |
| X00860 | Neohesperidin | 0.81 | 0.23 | 0.17 | 0.73 | 0.08 | 0.48 | 0.21 | 0.51 |
| X00865 | Poncirin | 0.22 | 0.06 | 0.16 | 0.06 | 0.04 | 0.17 | 0.05 | 0.11 |
| X00906 | Cysteinylglycine | 1.20 | 1.32 | 1.34 | 0.87 | 0.86 | 1.01 | 1.08 | 1.13 |
| X00907 | DL-Homocysteine | 0.02 | 0.01 | 0.02 | 0.03 | 0.06 | 0.05 | 0.01 | 0.01 |
| X00921 | Kaempferol-3-Rhamnoside-4"-Rhamnoside,-7-Rhamnoside | 0.16 | 0.24 | 0.22 | 0.15 | 0.63 | 0.25 | 0.11 | 0.21 |
| X00924 | Kaempferol-3-Glucuronide | 45.92 | 33.29 | 25.94 | 35.53 | 36.25 | 55.21 | 14.84 | 42.17 |
| X00933 | Quercetin-3-Glucuronide | 21.62 | 15.22 | 7.39 | 26.53 | 6.82 | 31.34 | 9.64 | 19.18 |
| X00943 | alpha-Methyl-DL-histidine dihydrochloride | 0.03 | 0.04 | 0.03 | 0.03 | 0.06 | 0.02 | 0.01 | 0.02 |
| X00971 | DL-5-Hydroxylysine hydrochloride | 0.01 | 0.01 | 0.03 | 0.04 | 0.02 | 0.02 | 0.01 | 0.03 |
| X00986 | L-Anserine | 0.04 | 0.05 | 0.03 | 0.04 | 0.04 | 0.04 | 0.04 | 0.04 |
| X01016 | Choline chloride | 31.55 | 26.68 | 26.95 | 30.70 | 32.46 | 42.35 | 31.97 | 22.65 |
| X01026 | Trigonelline hydrochloride | 4.35 | 1.75 | 0.77 | 3.45 | 3.28 | 2.29 | 1.30 | 1.45 |
| X01097 | DL-Pipecolinic acid | 3.56 | 2.65 | 6.95 | 3.33 | 5.93 | 5.16 | 3.27 | 4.91 |
| X01098 | Maleic acid | 0.06 | 0.12 | 0.05 | 0.05 | 0.15 | 0.14 | 0.06 | 0.03 |
| X01099 | D-(+)-Raffinose pentahydrate | 0.55 | 0.34 | 0.24 | 0.43 | 0.12 | 0.15 | 0.24 | 0.18 |
| X01144 | S-Methyl-L-cysteine | 0.53 | 0.43 | 0.55 | 0.49 | 0.67 | 1.07 | 0.93 | 0.38 |
| X01218 | Petunidin-3-O-beta-glucopyranoside | 3.61 | 3.80 | 1.29 | 5.76 | 2.25 | 3.26 | 5.22 | 3.04 |
| X01219 | Quercetin-3,4'-O-di-beta-glucopyranoside | 0.44 | 0.10 | 0.10 | 0.52 | 0.06 | 0.29 | 0.76 | 0.30 |
| X01220 | Cyanidin-3-O-(2"-O-beta-xylopyranosyl-beta-glucopyranoside) | 1.01 | 0.46 | 1.52 | 0.86 | 0.53 | 1.41 | 0.97 | 0.43 |
| X01223 | Delphinidin-3-O-(6"-O-alpha-rhamnopyranosyl-beta-glucopyranoside) | 3.24 | 1.39 | 0.85 | 3.46 | 0.60 | 3.30 | 1.01 | 2.41 |
| X01224 | Delphinidin-3-O-(2"-O-beta-xylopyranosyl-beta-glucopyranoside) | 0.27 | 0.23 | 0.09 | 0.45 | 0.11 | 0.26 | 0.46 | 0.27 |
| X01225 | Cyanidin-3-O-(2"-O-beta-xylopyranosyl-beta-glucopyranoside)-5-O-beta-glucopyranoside | 0.38 | 0.36 | 0.27 | 0.16 | 0.35 | 0.22 | 0.24 | 0.17 |
| X100024 | L-Leucine, (Cell Culture Reagent, Crystalline) | 0.97 | 0.43 | 0.63 | 1.02 | 1.26 | 0.76 | 1.85 | 0.60 |
| X100025 | L-Isoleucine | 0.62 | 0.39 | 0.48 | 0.73 | 1.02 | 0.61 | 1.24 | 0.48 |
| X100027 | Methionine sulfoxide | 0.13 | 0.09 | 0.18 | 0.19 | 0.16 | 0.17 | 0.54 | 0.17 |
| X100039 | Luteolin 7-O-glucuronide | 46.15 | 34.22 | 26.66 | 37.68 | 37.66 | 48.10 | 14.28 | 43.67 |
| X100043 | 2i_Leu_Ile | 7.67 | 4.27 | 5.33 | 8.88 | 11.21 | 6.19 | 15.23 | 5.18 |
| X100044 | 2i_Gln_Lys | 41.16 | 30.46 | 93.40 | 37.58 | 64.14 | 59.30 | 44.86 | 57.94 |

| | | | | | | | | | |
|---|---|---|---|---|---|---|---|---|---|
| X200004 | 3i_Isorhamnetin-3-O-rutinoside_Isorhamnetin-3-Glucoside-6"-Rhamnoside_Isorhamnetin-3-Galactoside-6"-Rhamnoside | 8.87 | 3.30 | 1.54 | 10.25 | 1.67 | 5.15 | 2.15 | 5.88 |
| X200007 | 2i_Cyanidin-3,5-di-O-glucoside chloride_Cyanidin-3-O-(2"-O-beta-glucopyranosyl-beta-glucopyranoside) | 5.93 | 3.38 | 6.36 | 6.28 | 5.56 | 7.04 | 8.16 | 4.10 |
| X200011 | 2i_Ideain chloride_Cyanidin-3-glucoside chloride | 22.02 | 11.02 | 26.27 | 25.31 | 21.74 | 32.89 | 32.52 | 20.11 |
| X200012 | 2i_Maritimein_luteolin-7-O-glucoside | 17.44 | 11.36 | 21.65 | 20.76 | 17.69 | 26.82 | 26.38 | 16.43 |
| X200017 | 2i_Syringetin-3-O-glucoside_Syringetin-3-O-galactoside | 1.31 | 0.00 | 0.43 | 3.13 | 0.00 | 0.01 | 1.89 | 1.28 |
| X200029 | 2i_alpha-D-Glucose-1-phosphate dipotassium salt dihydate_alpha-D-Galactose-1-phosphate dipotassium salt pentahydrate | 0.16 | 0.14 | 0.20 | 0.18 | 0.16 | 0.28 | 0.09 | 0.16 |
| X200030 | 2i_D-Mannose-6-phosphate barium salt hydrate_D-Mannose 6-phosphate mono sodium salt | 0.21 | 0.14 | 0.17 | 0.15 | 0.12 | 0.23 | 0.10 | 0.22 |
| X200031 | 4i_D-Fructose-6-phosphate disodium salt hydrate _D-Glucose-6-phosphate sodium salt _alpha-D-(+)-mannose-1-phosphate sodium salt hydrate_alpha-D(+)Mannose 1-phosphate bis(cyclohexylammonium) salt | 0.21 | 0.16 | 0.17 | 0.22 | 0.16 | 0.23 | 0.11 | 0.18 |
| X200034 | 2i_D(+)-Galactosamine hydrochloride_D-(+)-Glucosamine hydrochloride | 0.03 | 0.03 | 0.03 | 0.05 | 0.05 | 0.08 | 0.08 | 0.06 |
| X200042 | 2i_D-(+)-Cellobiose_Lactulose | 0.72 | 0.58 | 0.52 | 0.54 | 0.43 | 0.73 | 0.53 | 0.55 |
| X200043 | 6i_Melibiose hydrate_D-(+)-Turanose_Isomaltose_Gentiobiose_MelibiosePalatinose Monohydrate | 4.76 | 3.70 | 3.16 | 3.60 | 2.99 | 4.26 | 3.31 | 3.26 |
| X200047 | 4i_L(+)-Arginine HCl_N-alpha-Acetyl-L-ornithine_L-Citrulline | 1.48 | 1.91 | 14.12 | 7.27 | 49.06 | 2.73 | 8.29 | 4.56 |
| X200054 | DL-Cysteine | 0.02 | 0.02 | 0.02 | 0.01 | 0.02 | 0.01 | 0.02 | 0.02 |
| X200055 | 2i_Nicotinamide_Niacinamide | 0.51 | 0.33 | 0.40 | 0.39 | 0.46 | 0.93 | 0.62 | 0.28 |
| X200057 | 2i_L-Alanine_Sarcosine | 6.93 | 4.98 | 8.89 | 7.99 | 5.93 | 8.10 | 9.68 | 6.82 |
| X200060 | 2i_L-Norvaline_L-Valine | 3.36 | 2.92 | 4.09 | 3.52 | 6.17 | 4.06 | 6.10 | 3.89 |
| X200061 | 3i_L-Threonine_alpha-Methyl-DL-serine_L-Homoserine | 2.40 | 1.49 | 2.74 | 2.09 | 3.03 | 2.55 | 2.54 | 1.98 |
| X200063 | 3i_L-2-Aminobutyric acid_N,N-Dimethylglycine hydrochloride_N-Methyl-DL-Alanine | 5.91 | 5.41 | 5.24 | 5.69 | 6.32 | 8.06 | 6.22 | 4.33 |
| X200068 | DL-Malic acid | 2.51 | 6.87 | 2.47 | 2.49 | 7.30 | 4.84 | 2.16 | 1.65 |

| | | | | | | | | | |
|---|---|---|---|---|---|---|---|---|---|
| X200072 | L-CarnitineHCl | 0.03 | 0.01 | 0.02 | 0.04 | 0.05 | 0.02 | 0.02 | 0.04 |
| X200073 | DL-2-Aminoadipic Acid | 0.19 | 0.07 | 0.12 | 0.14 | 0.21 | 0.14 | 0.11 | 0.09 |
| X200082 | L-Lysine HCl | 41.67 | 30.93 | 95.22 | 38.34 | 64.44 | 59.44 | 45.69 | 58.55 |
| X200085 | 2i_L-Norleucine_D-Alloisoleucine | 7.54 | 3.98 | 4.96 | 8.70 | 11.37 | 6.39 | 14.54 | 5.06 |
| X200087 | 2i_Rutin _Quercetin-3-O-b-glucopyranosyl-7-O-a-rhamnopyranoside | 2.26 | 0.57 | 0.58 | 2.02 | 0.23 | 1.33 | 0.58 | 1.43 |
| X200089 | 2i_D-(+)-Melezitose monohydrate_1-Kestose | 0.52 | 0.23 | 0.20 | 0.42 | 0.12 | 0.19 | 0.25 | 0.18 |
| X260002 | gamma-Glu-PRENCSO | 1.27 | 0.30 | 1.46 | 0.66 | 0.87 | 1.04 | 0.93 | 0.56 |
| X260006 | MCSO | 1.05 | 0.86 | 3.85 | 0.98 | 1.55 | 9.61 | 6.47 | 0.73 |
| X260007 | Alliin | 39.55 | 18.79 | 37.95 | 22.57 | 27.00 | 30.74 | 31.45 | 30.19 |

| **ID** | **Annotation** | **CHN180** | **GRE307** | **EGY489** | **CHN524** | **TUR542** | **GER556** | **F115** | **F138** |
|---|---|---|---|---|---|---|---|---|---|
| X00004 | 1-Amino-1-cyclopentanecarboxylic acid | 4.02 | 5.80 | 4.80 | 4.10 | 6.87 | 3.52 | 3.51 | 4.00 |
| X00010 | Citric acid,Anhydrous | 1.66 | 6.53 | 1.51 | 2.40 | 4.47 | 0.90 | 3.59 | 1.35 |
| X00039 | Adenine | 1.42 | 1.61 | 0.44 | 0.68 | 0.99 | 0.85 | 0.84 | 0.95 |
| X00049 | Glycine | 0.32 | 0.26 | 0.16 | 0.08 | 0.17 | 0.30 | 0.18 | 0.43 |
| X00054 | L-(-)-Phenylalanine | 2.69 | 1.63 | 1.68 | 0.89 | 2.07 | 3.62 | 1.35 | 3.40 |
| X00055 | L-Tryptophane | 3.97 | 2.32 | 0.28 | 0.47 | 1.15 | 4.00 | 0.50 | 2.13 |
| X00056 | L-Tyrosine | 1.28 | 1.10 | 0.15 | 0.31 | 0.44 | 0.73 | 0.34 | 0.60 |
| X00060 | Sucrose | 5.02 | 5.85 | 4.73 | 3.17 | 6.85 | 7.30 | 4.25 | 7.77 |
| X00095 | Methylmalonic acid | 0.38 | 0.32 | 0.34 | 0.42 | 0.50 | 0.37 | 0.30 | 0.59 |
| X00099 | Pyridoxamine dihydrochloride | 0.07 | 0.12 | 0.06 | 0.07 | 0.05 | 0.06 | 0.16 | 0.09 |
| X00187 | Cystathionine | 0.01 | 0.01 | 0.01 | 0.02 | 0.01 | 0.01 | 0.01 | 0.01 |
| X00188 | Cytidine,cell culture tested | 1.89 | 6.30 | 0.85 | 0.90 | 1.85 | 1.36 | 1.06 | 1.30 |
| X00212 | DL-2,3-Diaminopropionic acid monohydrochloride | 0.05 | 0.25 | 0.08 | 0.16 | 0.16 | 0.21 | 0.12 | 0.25 |
| X00218 | DL-threo-beta-Methylaspartic acid | 0.05 | 0.10 | 0.06 | 0.06 | 0.11 | 0.02 | 0.05 | 0.06 |
| X00241 | Guanosine | 0.31 | 0.26 | 0.50 | 0.24 | 0.27 | 0.24 | 0.18 | 0.32 |
| X00256 | Inosine | 0.13 | 0.12 | 0.36 | 0.14 | 0.12 | 0.12 | 0.07 | 0.19 |
| X00267 | L-Asparagine | 2.97 | 3.20 | 4.44 | 3.24 | 3.78 | 1.18 | 1.39 | 1.40 |
| X00268 | L-Aspartic acid | 3.31 | 4.35 | 4.06 | 4.01 | 4.93 | 2.63 | 3.22 | 3.45 |
| X00270 | L-Cystine | 0.04 | 0.07 | 0.05 | 0.02 | 0.04 | 0.14 | 0.02 | 0.12 |
| X00272 | L-Glutamic acid | 37.38 | 32.97 | 34.47 | 25.38 | 42.93 | 28.49 | 24.15 | 39.71 |
| X00273 | L-Glutamine | 27.07 | 28.12 | 22.30 | 27.41 | 23.18 | 15.97 | 16.45 | 20.39 |
| X00285 | L-Ornithine monohydrochloride | 0.71 | 0.78 | 0.89 | 0.82 | 0.64 | 0.77 | 0.36 | 0.36 |
| X00286 | L-Proline | 16.36 | 15.24 | 9.06 | 6.23 | 9.41 | 13.12 | 5.80 | 14.70 |
| X00287 | L-Pyroglutamic acid | 4.35 | 6.23 | 5.30 | 4.64 | 7.19 | 3.72 | 3.65 | 4.40 |
| X00288 | L-Serine | 6.16 | 7.16 | 7.32 | 5.23 | 8.53 | 3.08 | 3.18 | 7.03 |

| | | | | | | | | | |
|---|---|---|---|---|---|---|---|---|---|
| X00338 | S-Carboxymethyl-L-cysteine | 0.14 | 0.08 | 0.13 | 0.10 | 0.14 | 0.12 | 0.08 | 0.10 |
| X00344 | Succinic acid | 0.35 | 0.29 | 0.33 | 0.44 | 0.51 | 0.41 | 0.33 | 0.59 |
| X00364 | Tyramine | 0.81 | 0.57 | 1.00 | 0.97 | 1.10 | 0.22 | 0.69 | 0.48 |
| X00372 | Uridine-5'-monophosphate | 0.03 | 0.02 | 0.02 | 0.03 | 0.05 | 0.05 | 0.03 | 0.03 |
| X00379 | alpha-Lactose monohydrate | 0.52 | 0.57 | 0.40 | 0.33 | 0.62 | 0.62 | 0.39 | 0.73 |
| X00389 | gamma-Amino-n-butyric acid | 1.34 | 4.62 | 1.92 | 3.40 | 3.22 | 4.28 | 2.66 | 4.67 |
| X00392 | 6-Aminohexanoic acid | 1.88 | 1.22 | 0.30 | 0.36 | 0.73 | 0.87 | 0.39 | 0.77 |
| X00416 | Keracyanin Chloride | 0.87 | 3.99 | 3.60 | 2.33 | 4.10 | 2.08 | 1.59 | 3.48 |
| X00419 | Isorhamnetin-3-O-glucoside | 8.30 | 16.75 | 0.84 | 3.28 | 3.34 | 1.88 | 0.86 | 1.14 |
| X00422 | Kaempferol-3-O-glucoside | 18.00 | 21.91 | 6.01 | 7.43 | 17.11 | 10.43 | 4.08 | 6.45 |
| X00432 | Hyperoside | 5.15 | 14.61 | 0.51 | 2.72 | 3.74 | 1.32 | 0.80 | 0.65 |
| X00477 | O-Acetyl-L-serine hydrochloride | 0.47 | 0.41 | 0.52 | 0.43 | 0.69 | 0.22 | 0.43 | 0.40 |
| X00502 | L-Saccharopine | 0.51 | 0.52 | 0.10 | 0.18 | 0.27 | 0.27 | 0.15 | 0.17 |
| X00675 | 5'-Deoxy-5'-Methylthioadenosine | 1.37 | 2.28 | 1.48 | 1.10 | 2.80 | 0.97 | 1.54 | 1.36 |
| X00695 | L-allo-threonine | 2.84 | 2.58 | 1.93 | 1.41 | 2.28 | 2.17 | 1.14 | 2.18 |
| X00755 | 5-Aminovaleric acid | 4.24 | 3.54 | 1.82 | 1.43 | 2.23 | 2.63 | 1.59 | 3.20 |
| X00797 | L-Threonic acid hemicalcium salt | 0.76 | 0.69 | 0.67 | 0.71 | 1.02 | 0.74 | 1.11 | 0.75 |
| X00849 | Hesperidin | 0.17 | 0.36 | 0.20 | 0.31 | 0.40 | 0.02 | 0.13 | 0.08 |
| X00853 | Luteolin-3',7-di-O-glucoside | 10.48 | 13.72 | 2.97 | 4.23 | 8.03 | 2.85 | 2.51 | 1.85 |
| X00854 | Luteolin-4'-O-glucoside | 40.61 | 50.48 | 13.33 | 17.93 | 37.50 | 21.18 | 9.82 | 13.20 |
| X00860 | Neohesperidin | 0.34 | 0.75 | 0.46 | 0.64 | 0.81 | 0.10 | 0.25 | 0.15 |
| X00865 | Poncirin | 0.17 | 0.18 | 0.12 | 0.10 | 0.17 | 0.06 | 0.01 | 0.10 |
| X00906 | Cysteinylglycine | 1.28 | 0.84 | 0.97 | 0.95 | 0.43 | 1.14 | 0.79 | 0.92 |
| X00907 | DL-Homocysteine | 0.01 | 0.00 | 0.02 | 0.01 | 0.02 | 0.03 | 0.02 | 0.04 |
| X00921 | Kaempferol-3-Rhamnoside-4"-Rhamnoside,-7-Rhamnoside | 0.12 | 0.09 | 0.17 | 0.15 | 0.20 | 0.09 | 0.22 | 0.19 |
| X00924 | Kaempferol-3-Glucuronide | 15.10 | 33.19 | 44.22 | 35.21 | 53.75 | 29.40 | 53.39 | 52.81 |
| X00933 | Quercetin-3-Glucuronide | 8.60 | 26.34 | 25.21 | 24.12 | 26.49 | 9.16 | 35.96 | 22.94 |
| X00943 | alpha-Methyl-DL-histidine dihydrochloride | 0.03 | 0.01 | 0.02 | 0.02 | 0.03 | 0.01 | 0.01 | 0.02 |
| X00971 | DL-5-Hydroxylysine hydrochloride | 0.02 | 0.02 | 0.01 | 0.02 | 0.02 | 0.01 | 0.00 | 0.00 |
| X00986 | L-Anserine | 0.05 | 0.04 | 0.06 | 0.04 | 0.02 | 0.04 | 0.04 | 0.05 |
| X01016 | Choline chloride | 28.90 | 36.84 | 28.60 | 21.19 | 39.10 | 21.83 | 28.85 | 29.45 |
| X01026 | Trigonelline hydrochloride | 2.12 | 4.83 | 1.29 | 1.63 | 2.17 | 2.17 | 1.87 | 2.10 |
| X01097 | DL-Pipecolinic acid | 3.16 | 3.70 | 2.83 | 4.05 | 3.93 | 3.14 | 2.73 | 2.90 |
| X01098 | Maleic acid | 0.07 | 0.08 | 0.05 | 0.04 | 0.10 | 0.03 | 0.11 | 0.07 |
| X01099 | D-(+)-Raffinose pentahydrate | 0.24 | 0.37 | 0.14 | 0.24 | 0.38 | 1.50 | 0.19 | 0.57 |
| X01144 | S-Methyl-L-cysteine | 0.90 | 1.28 | 0.38 | 0.55 | 0.66 | 0.63 | 0.65 | 0.75 |
| X01218 | Petunidin-3-O-beta-glucopyranoside | 6.44 | 7.17 | 2.76 | 4.05 | 7.15 | 3.62 | 8.64 | 3.58 |
| X01219 | Quercetin-3,4'-O-di-beta-glucopyranoside | 0.68 | 1.64 | 0.20 | 0.32 | 0.56 | 0.14 | 0.39 | 0.10 |
| X01220 | Cyanidin-3-O-(2"-O-beta-xylopyranosyl-beta-glucopyranoside) | 1.29 | 2.09 | 0.35 | 0.49 | 1.24 | 0.94 | 0.36 | 0.38 |
| X01223 | Delphinidin-3-O-(6"-O-alpha-rhamnopyranosyl-beta-glucopyranoside) | 1.27 | 4.52 | 3.23 | 4.04 | 4.23 | 0.77 | 2.36 | 1.66 |
| X01224 | Delphinidin-3-O-(2"-O-beta-xylopyranosyl-beta-glucopyranoside) | 0.51 | 0.64 | 0.24 | 0.34 | 0.53 | 0.21 | 0.45 | 0.19 |

| | | | | | | | | | |
|---|---|---|---|---|---|---|---|---|---|
| X01225 | Cyanidin-3-O-(2"-O-beta-xylopyranosyl-beta-glucopyranoside)-5-O-beta-glucopyranoside | 0.26 | 0.30 | 0.13 | 0.12 | 0.42 | 0.38 | 0.50 | 0.33 |
| X100024 | L-Leucine, (Cell Culture Reagent, Crystalline) | 2.59 | 1.91 | 0.31 | 0.48 | 0.97 | 1.23 | 0.51 | 0.94 |
| X100025 | L-Isoleucine | 1.83 | 1.29 | 0.28 | 0.39 | 0.69 | 0.88 | 0.40 | 0.79 |
| X100027 | Methionine sulfoxide | 0.68 | 0.43 | 0.07 | 0.13 | 0.20 | 0.34 | 0.10 | 0.26 |
| X100039 | Luteolin 7-O-glucuronide | 15.56 | 35.04 | 45.72 | 35.56 | 56.98 | 30.18 | 54.75 | 53.02 |
| X100043 | 2i_Leu_Ile | 21.96 | 15.49 | 2.95 | 4.08 | 8.43 | 10.28 | 4.36 | 8.48 |
| X100044 | 2i_Gln_Lys | 50.53 | 52.89 | 39.60 | 48.67 | 42.99 | 31.53 | 29.45 | 37.67 |
| X200004 | 3i_Isorhamnetin-3-O-rutinoside_Isorhamnetin-3-Glucoside-6"-Rhamnoside_Isorhamnetin-3-Galactoside-6"-Rhamnoside | 2.61 | 9.11 | 8.62 | 9.54 | 7.41 | 2.15 | 4.96 | 5.15 |
| X200007 | 2i_Cyanidin-3,5-di-O-glucoside chloride_Cyanidin-3-O-(2"-O-beta-glucopyranosyl-beta-glucopyranoside) | 10.84 | 14.47 | 3.21 | 4.09 | 8.17 | 3.05 | 2.60 | 1.79 |
| X200011 | 2i_Ideain chloride_Cyanidin-3-glucoside chloride | 38.64 | 47.69 | 12.19 | 17.05 | 36.64 | 21.24 | 10.21 | 8.95 |
| X200012 | 2i_Maritimein_luteolin-7-O-glucoside | 31.66 | 40.16 | 10.51 | 13.32 | 30.51 | 17.22 | 7.48 | 10.28 |
| X200017 | 2i_Syringetin-3-O-glucoside_Syringetin-3-O-galactoside | 1.17 | 2.22 | 1.37 | 1.86 | 2.70 | 0.11 | 0.17 | 0.06 |
| X200029 | 2i_alpha-D-Glucose-1-phosphate dipotassium salt dihydate_alpha-D-Galactose-1-phosphate dipotassium salt pentahydrate | 0.22 | 0.08 | 0.21 | 0.13 | 0.22 | 0.09 | 0.10 | 0.11 |
| X200030 | 2i_D-Mannose-6-phosphate barium salt hydrate_D-Mannose 6-phosphate mono sodium salt | 0.26 | 0.08 | 0.16 | 0.23 | 0.21 | 0.03 | 0.09 | 0.11 |
| X200031 | 4i_D-Fructose-6-phosphate disodium salt hydrate _D-Glucose-6-phosphate sodium salt _alpha-D-(+)-mannose-1-phosphate sodium salt hydrate_alpha-D(+)Mannose 1-phosphate bis(cyclohexylammonium) salt | 0.23 | 0.08 | 0.16 | 0.26 | 0.20 | 0.08 | 0.14 | 0.11 |
| X200034 | 2i_D(+)-Galactosamine hydrochloride_D-(+)-Glucosamine hydrochloride | 0.08 | 0.05 | 0.04 | 0.02 | 0.04 | 0.04 | 0.03 | 0.04 |
| X200042 | 2i_D-(+)-Cellobiose_Lactulose | 0.58 | 0.57 | 0.46 | 0.33 | 0.74 | 0.80 | 0.40 | 0.79 |
| X200043 | 6i_Melibiose hydrate_D-(+)-Turanose_Isomaltose_Gentiobiose_MelibiosePalatinose Monohydrate | 3.42 | 3.74 | 2.91 | 2.02 | 4.40 | 4.65 | 2.77 | 5.03 |
| X200047 | 4i_L(+)-Arginine HCl_N-alpha-Acetyl-L-ornithine_L-Citrulline | 7.83 | 16.35 | 4.74 | 3.98 | 3.95 | 15.10 | 1.28 | 2.03 |

| | | | | | | | | | |
|---|---|---|---|---|---|---|---|---|---|
| X200054 | DL-Cysteine | 0.02 | 0.01 | 0.01 | 0.01 | 0.04 | 0.02 | 0.01 | 0.03 |
| X200055 | 2i_Nicotinamide_Niacinamide | 0.50 | 0.84 | 0.36 | 0.36 | 0.61 | 0.30 | 0.48 | 0.44 |
| X200057 | 2i_L-Alanine_Sarcosine | 9.91 | 7.92 | 6.20 | 5.25 | 8.05 | 7.22 | 6.12 | 7.65 |
| X200060 | 2i_L-Norvaline_L-Valine | 6.79 | 5.48 | 2.94 | 2.30 | 3.43 | 4.23 | 2.78 | 5.41 |
| X200061 | 3i_L-Threonine_alpha-Methyl-DL-serine_L-Homoserine | 2.84 | 2.23 | 1.71 | 1.18 | 2.31 | 1.84 | 1.20 | 2.14 |
| X200063 | 3i_L-2-Aminobutyric acid_N,N-Dimethylglycine hydrochloride_N-Methyl-DL-Alanine | 5.84 | 7.14 | 5.84 | 4.27 | 7.66 | 4.26 | 5.84 | 5.69 |
| X200068 | DL-Malic acid | 2.66 | 2.83 | 2.73 | 2.28 | 3.73 | 1.61 | 6.62 | 2.92 |
| X200072 | L-CarnitineHCl | 0.03 | 0.01 | 0.03 | 0.03 | 0.02 | 0.02 | 0.03 | 0.03 |
| X200073 | DL-2-Aminoadipic Acid | 0.11 | 0.08 | 0.08 | 0.06 | 0.12 | 0.09 | 0.05 | 0.08 |
| X200082 | L-Lysine HCl | 51.60 | 53.53 | 40.00 | 48.73 | 43.84 | 31.71 | 29.25 | 37.78 |
| X200085 | 2i_L-Norleucine_D-Alloisoleucine | 21.32 | 15.74 | 2.90 | 3.75 | 7.40 | 10.61 | 4.19 | 8.07 |
| X200087 | 2i_Rutin _Quercetin-3-O-b-glucopyranosyl-7-O-a-rhamnopyranoside | 1.01 | 2.34 | 1.35 | 1.87 | 2.07 | 0.29 | 0.79 | 0.56 |
| X200089 | 2i_D-(+)-Melezitose monohydrate_1-Kestose | 0.20 | 0.39 | 0.16 | 0.20 | 0.36 | 1.52 | 0.13 | 0.54 |
| X260002 | gamma-Glu-PRENCSO | 0.63 | 0.40 | 0.49 | 0.08 | 0.63 | 0.78 | 0.10 | 0.92 |
| X260006 | MCSO | 1.11 | 1.17 | 2.35 | 2.02 | 4.08 | 13.60 | 9.99 | 14.04 |
| X260007 | Alliin | 25.56 | 17.67 | 24.13 | 18.01 | 25.42 | 23.08 | 14.24 | 25.09 |
| **ID** | **Annotation** | **F189** | **F1-200-34** | **Fs407** | **Fs414** | **Fs422** | **SAN BARTOROME（Gatur）** | **SYR_No. 5** | **IND-IIT** |
| X00004 | 1-Amino-1-cyclopentanecarboxylic acid | 3.55 | 5.70 | 2.27 | 5.23 | 3.83 | 5.36 | 6.08 | 11.17 |
| X00010 | Citric acid,Anhydrous | 1.29 | 3.03 | 1.71 | 4.15 | 2.41 | 3.71 | 0.41 | 3.92 |
| X00039 | Adenine | 0.74 | 0.85 | 0.67 | 0.76 | 0.70 | 1.32 | 0.39 | 1.73 |
| X00049 | Glycine | 0.56 | 0.61 | 0.28 | 0.14 | 0.08 | 0.24 | 0.13 | 0.48 |
| X00054 | L-(-)-Phenylalanine | 6.59 | 5.15 | 6.36 | 2.42 | 1.37 | 2.71 | 1.46 | 2.55 |
| X00055 | L-Tryptophane | 2.96 | 2.99 | 1.64 | 1.06 | 0.26 | 2.16 | 0.31 | 3.78 |
| X00056 | L-Tyrosine | 0.87 | 0.83 | 0.53 | 0.41 | 0.21 | 0.79 | 0.20 | 1.38 |
| X00060 | Sucrose | 7.42 | 7.56 | 7.08 | 5.22 | 2.92 | 5.60 | 3.84 | 8.37 |
| X00095 | Methylmalonic acid | 0.28 | 0.59 | 0.38 | 0.49 | 0.40 | 0.36 | 0.29 | 0.33 |
| X00099 | Pyridoxamine dihydrochloride | 0.12 | 0.13 | 0.06 | 0.08 | 0.04 | 0.18 | 0.11 | 0.11 |

| | | | | | | | | | |
|---|---|---|---|---|---|---|---|---|---|
| X00187 | Cystathionine | 0.02 | 0.02 | 0.02 | 0.02 | 0.02 | 0.03 | 0.01 | 0.01 |
| X00188 | Cytidine,cell culture tested | 1.54 | 3.25 | 1.07 | 1.99 | 0.88 | 3.57 | 0.81 | 5.07 |
| X00212 | DL-2,3-Diaminopropionic acid monohydrochloride | 0.13 | 0.24 | 0.17 | 0.12 | 0.09 | 0.10 | 0.04 | 0.20 |
| X00218 | DL-threo-beta-Methylaspartic acid | 0.05 | 0.08 | 0.04 | 0.04 | 0.06 | 0.06 | 0.05 | 0.10 |
| X00241 | Guanosine | 0.13 | 0.52 | 0.30 | 0.45 | 0.24 | 0.34 | 0.30 | 0.66 |
| X00256 | Inosine | 0.07 | 0.37 | 0.13 | 0.23 | 0.15 | 0.20 | 0.20 | 0.28 |
| X00267 | L-Asparagine | 2.49 | 5.05 | 2.18 | 1.93 | 1.40 | 3.59 | 5.03 | 4.13 |
| X00268 | L-Aspartic acid | 3.52 | 5.76 | 2.23 | 5.49 | 3.54 | 6.15 | 3.34 | 3.72 |
| X00270 | L-Cystine | 0.07 | 0.15 | 0.08 | 0.10 | 0.02 | 0.02 | 0.06 | 0.11 |
| X00272 | L-Glutamic acid | 33.72 | 37.63 | 25.13 | 36.44 | 22.13 | 42.17 | 32.92 | 49.66 |
| X00273 | L-Glutamine | 36.63 | 37.43 | 20.66 | 27.27 | 15.08 | 36.21 | 38.37 | 57.37 |
| X00285 | L-Ornithine monohydrochloride | 0.69 | 1.65 | 0.60 | 0.46 | 0.28 | 0.80 | 1.07 | 0.99 |
| X00286 | L-Proline | 13.86 | 12.18 | 8.67 | 9.19 | 4.16 | 14.83 | 10.09 | 30.70 |
| X00287 | L-Pyroglutamic acid | 3.89 | 7.59 | 2.52 | 5.80 | 3.97 | 5.64 | 7.00 | 12.58 |
| X00288 | L-Serine | 6.70 | 5.77 | 4.11 | 6.16 | 2.76 | 9.24 | 5.02 | 7.02 |
| X00338 | S-Carboxymethyl-L-cysteine | 0.09 | 0.16 | 0.09 | 0.16 | 0.09 | 0.14 | 0.07 | 0.12 |
| X00344 | Succinic acid | 0.25 | 0.60 | 0.36 | 0.51 | 0.39 | 0.40 | 0.32 | 0.32 |
| X00364 | Tyramine | 0.34 | 0.42 | 0.89 | 0.19 | 0.14 | 1.06 | 0.69 | 0.69 |
| X00372 | Uridine-5'-monophosphate | 0.03 | 0.03 | 0.03 | 0.02 | 0.02 | 0.04 | 0.02 | 0.04 |
| X00379 | alpha-Lactose monohydrate | 0.68 | 0.69 | 0.62 | 0.40 | 0.26 | 0.57 | 0.32 | 0.74 |
| X00389 | gamma-Amino-n-butyric acid | 2.74 | 4.96 | 4.26 | 2.72 | 1.91 | 2.74 | 1.22 | 3.98 |
| X00392 | 6-Aminohexanoic acid | 1.19 | 1.20 | 0.73 | 0.73 | 0.24 | 1.13 | 0.32 | 1.49 |
| X00416 | Keracyanin Chloride | 2.55 | 3.01 | 2.01 | 3.54 | 1.22 | 2.41 | 1.59 | 1.71 |
| X00419 | Isorhamnetin-3-O-glucoside | 2.15 | 1.28 | 1.12 | 1.14 | 1.30 | 7.62 | 0.50 | 2.85 |
| X00422 | Kaempferol-3-O-glucoside | 10.68 | 6.46 | 6.22 | 10.37 | 13.94 | 16.41 | 8.18 | 26.77 |
| X00432 | Hyperoside | 1.16 | 0.65 | 0.80 | 0.73 | 0.13 | 6.00 | 0.35 | 3.60 |
| X00477 | O-Acetyl-L-serine hydrochloride | 0.36 | 0.33 | 0.33 | 0.53 | 0.52 | 0.41 | 0.38 | 0.44 |
| X00502 | L-Saccharopine | 0.29 | 0.23 | 0.19 | 0.18 | 0.12 | 0.50 | 0.12 | 0.58 |
| X00675 | 5'-Deoxy-5'-Methylthioadenosine | 1.23 | 2.47 | 1.06 | 2.45 | 1.91 | 1.94 | 0.85 | 2.48 |
| X00695 | L-allo-threonine | 2.42 | 3.04 | 1.82 | 2.35 | 0.83 | 2.96 | 1.79 | 3.58 |
| X00755 | 5-Aminovaleric acid | 3.95 | 3.69 | 3.20 | 2.66 | 1.09 | 3.62 | 1.64 | 4.14 |
| X00797 | L-Threonic acid hemicalcium salt | 0.67 | 0.89 | 0.84 | 1.11 | 0.86 | 0.88 | 0.46 | 1.12 |
| X00849 | Hesperidin | 0.04 | 0.05 | 0.16 | 0.12 | 0.06 | 0.18 | 0.04 | 0.13 |
| X00853 | Luteolin-3',7-di-O-glucoside | 3.60 | 3.06 | 1.83 | 4.76 | 5.97 | 8.33 | 3.82 | 10.14 |
| X00854 | Luteolin-4'-O-glucoside | 20.80 | 22.62 | 13.10 | 31.93 | 47.60 | 37.19 | 18.37 | 59.43 |
| X00860 | Neohesperidin | 0.09 | 0.13 | 0.32 | 0.21 | 0.10 | 0.41 | 0.14 | 0.27 |
| X00865 | Poncirin | 0.11 | 0.09 | 0.06 | 0.13 | 0.01 | 0.12 | 0.05 | 0.01 |
| X00906 | Cysteinylglycine | 0.85 | 1.43 | 1.03 | 1.59 | 0.79 | 1.66 | 0.77 | 1.22 |
| X00907 | DL-Homocysteine | 0.02 | 0.10 | 0.03 | 0.02 | 0.02 | 0.03 | 0.02 | 0.05 |
| X00921 | Kaempferol-3-Rhamnoside-4''-Rhamnoside,-7-Rhamnoside | 0.19 | 0.13 | 0.12 | 0.25 | 0.23 | 0.17 | 0.11 | 0.42 |
| X00924 | Kaempferol-3-Glucuronide | 45.52 | 43.00 | 32.21 | 69.91 | 27.64 | 34.28 | 17.59 | 57.96 |
| X00933 | Quercetin-3-Glucuronide | 16.92 | 15.90 | 26.38 | 19.41 | 18.58 | 15.20 | 7.89 | 28.49 |
| X00943 | alpha-Methyl-DL-histidine dihydrochloride | 0.02 | 0.04 | 0.01 | 0.02 | 0.02 | 0.03 | 0.02 | 0.02 |
| X00971 | DL-5-Hydroxylysine hydrochloride | 0.02 | 0.01 | 0.01 | 0.01 | 0.04 | 0.02 | 0.01 | 0.02 |

| | | | | | | | | | |
|---|---|---|---|---|---|---|---|---|---|
| X00986 | L-Anserine | 0.04 | 0.03 | 0.06 | 0.04 | 0.03 | 0.07 | 0.04 | 0.05 |
| X01016 | Choline chloride | 26.02 | 32.47 | 23.21 | 37.13 | 28.95 | 31.15 | 19.33 | 41.05 |
| X01026 | Trigonelline hydrochloride | 2.34 | 3.16 | 1.65 | 1.90 | 0.95 | 2.17 | 1.28 | 4.32 |
| X01097 | DL-Pipecolinic acid | 5.52 | 5.45 | 1.92 | 3.91 | 2.77 | 5.50 | 6.01 | 9.16 |
| X01098 | Maleic acid | 0.03 | 0.09 | 0.06 | 0.11 | 0.06 | 0.07 | 0.05 | 0.11 |
| X01099 | D-(+)-Raffinose pentahydrate | 0.68 | 0.53 | 0.80 | 0.31 | 0.20 | 0.25 | 0.07 | 0.53 |
| X01144 | S-Methyl-L-cysteine | 0.65 | 1.13 | 0.70 | 0.69 | 0.60 | 0.82 | 0.52 | 1.20 |
| X01218 | Petunidin-3-O-beta-glucopyranoside | 4.30 | 3.61 | 5.11 | 9.24 | 43.08 | 5.92 | 1.19 | 7.54 |
| X01219 | Quercetin-3,4'-O-di-beta-glucopyranoside | 0.15 | 0.16 | 0.26 | 0.37 | 0.47 | 0.70 | 0.09 | 0.27 |
| X01220 | Cyanidin-3-O-(2"-O-beta-xylopyranosyl-beta-glucopyranoside) | 0.61 | 0.84 | 0.29 | 1.06 | 1.50 | 0.97 | 0.74 | 2.43 |
| X01223 | Delphinidin-3-O-(6"-O-alpha-rhamnopyranosyl-beta-glucopyranoside) | 1.00 | 1.12 | 2.60 | 1.77 | 1.48 | 2.44 | 0.67 | 2.04 |
| X01224 | Delphinidin-3-O-(2"-O-beta-xylopyranosyl-beta-glucopyranoside) | 0.21 | 0.24 | 0.29 | 0.42 | 2.19 | 0.50 | 0.09 | 0.60 |
| X01225 | Cyanidin-3-O-(2"-O-beta-xylopyranosyl-beta-glucopyranoside)-5-O-beta-glucopyranoside | 0.46 | 0.49 | 0.32 | 1.11 | 1.94 | 0.28 | 0.11 | 0.67 |
| X100024 | L-Leucine, (Cell Culture Reagent, Crystalline) | 1.57 | 1.54 | 0.85 | 0.97 | 0.32 | 1.68 | 0.38 | 2.50 |
| X100025 | L-Isoleucine | 1.21 | 1.13 | 0.63 | 0.65 | 0.22 | 1.15 | 0.29 | 1.46 |
| X100027 | Methionine sulfoxide | 0.36 | 0.39 | 0.22 | 0.15 | 0.06 | 0.48 | 0.08 | 0.50 |
| X100039 | Luteolin 7-O-glucuronide | 48.33 | 44.03 | 32.29 | 72.63 | 29.03 | 35.60 | 17.94 | 40.23 |
| X100043 | 2i_Leu_Ile | 13.33 | 13.10 | 7.59 | 8.17 | 2.59 | 14.31 | 3.40 | 19.75 |
| X100044 | 2i_Gln_Lys | 65.76 | 68.33 | 36.04 | 48.97 | 27.77 | 65.66 | 68.75 | 104.25 |
| X200004 | 3i_Isorhamnetin-3-O-rutinoside_Isorhamnetin-3-Glucoside-6"-Rhamnoside_Isorhamnetin-3-Galactoside-6"-Rhamnoside | 3.69 | 2.99 | 5.35 | 4.87 | 4.07 | 4.56 | 1.24 | 2.97 |
| X200007 | 2i_Cyanidin-3,5-di-O-glucoside chloride_Cyanidin-3-O-(2"-O-beta-glucopyranosyl-beta-glucopyranoside) | 3.51 | 3.72 | 1.75 | 4.81 | 6.15 | 8.26 | 3.82 | 10.01 |
| X200011 | 2i_Ideain chloride_Cyanidin-3-glucoside chloride | 19.28 | 21.09 | 11.34 | 23.35 | 45.84 | 35.69 | 16.95 | 56.95 |
| X200012 | 2i_Maritimein_luteolin-7-O-glucoside | 18.16 | 16.77 | 10.26 | 19.18 | 24.12 | 28.36 | 14.25 | 45.85 |
| X200017 | 2i_Syringetin-3-O-glucoside_Syringetin-3-O-galactoside | 0.07 | 0.22 | 0.21 | 0.09 | 0.19 | 1.71 | 0.01 | 0.01 |
| X200029 | 2i_alpha-D-Glucose-1-phosphate dipotassium salt dihydate_alpha-D-Galactose-1-phosphate dipotassium salt pentahydrate | 0.06 | 0.10 | 0.09 | 0.14 | 0.09 | 0.22 | 0.14 | 0.15 |

| | | | | | | | | | |
|---|---|---|---|---|---|---|---|---|---|
| X200030 | 2i_D-Mannose-6-phosphate bariumsalt hydrate_D-Mannose 6-phosphate mono sodiumsalt | 0.11 | 0.07 | 0.12 | 0.12 | 0.03 | 0.21 | 0.10 | 0.14 |
| X200031 | 4i_D-Fructose-6-phosphate disodiumsalt hydrate _D-Glucose-6-phosphate sodiumsalt _alpha-D-(+)-mannose-1-phosphate sodiumsalt hydrate_alpha-D(+)Mannose 1-phosphate bis(cyclohexylammonium) salt | 0.12 | 0.06 | 0.12 | 0.16 | 0.09 | 0.23 | 0.11 | 0.13 |
| X200034 | 2i_D(+)-Galactosamine hydrochloride_D-(+)-Glucosamine hydrochloride | 0.05 | 0.04 | 0.04 | 0.03 | 0.03 | 0.03 | 0.08 | 0.07 |
| X200042 | 2i_D-(+)-Cellobiose_Lactulose | 0.82 | 0.80 | 0.70 | 0.50 | 0.29 | 0.63 | 0.35 | 0.91 |
| X200043 | 6i_Melibiose hydrate_D-(+)-Turanose_Isomaltose_Gentiobiose_MelibiosePalatinose Monohydrate | 4.60 | 5.08 | 4.52 | 3.31 | 1.81 | 3.61 | 2.30 | 5.69 |
| X200047 | 4i_L(+)-Arginine HCl_N-alpha-Acetyl-L-ornithine_L-Citrulline | 6.80 | 48.19 | 2.24 | 5.54 | 2.17 | 7.08 | 12.71 | 9.95 |
| X200054 | DL-Cysteine | 0.01 | 0.03 | 0.01 | 0.03 | 0.02 | 0.02 | 0.02 | 0.03 |
| X200055 | 2i_Nicotinamide_Niacinamide | 0.39 | 0.41 | 0.36 | 0.64 | 0.29 | 0.68 | 0.23 | 0.72 |
| X200057 | 2i_L-Alanine_Sarcosine | 7.00 | 9.80 | 7.16 | 5.48 | 6.59 | 7.02 | 5.78 | 11.16 |
| X200060 | 2i_L-Norvaline_L-Valine | 6.40 | 6.30 | 5.26 | 4.12 | 1.90 | 6.10 | 2.81 | 6.78 |
| X200061 | 3i_L-Threonine_alpha-Methyl-DL-serine_L-Homoserine | 2.41 | 3.03 | 1.53 | 2.14 | 0.86 | 2.70 | 1.66 | 3.57 |
| X200063 | 3i_L-2-Aminobutyric acid_N,N-Dimethylglycine hydrochloride_N-Methyl-DL-Alanine | 5.10 | 6.64 | 4.42 | 7.19 | 5.69 | 6.09 | 4.06 | 8.42 |
| X200068 | DL-Malic acid | 2.28 | 3.52 | 3.54 | 5.68 | 2.76 | 3.04 | 1.93 | 4.38 |
| X200072 | L-CarnitineHCl | 0.04 | 0.04 | 0.04 | 0.03 | 0.03 | 0.02 | 0.04 | 0.12 |
| X200073 | DL-2-Aminoadipic Acid | 0.07 | 0.09 | 0.03 | 0.05 | 0.02 | 0.09 | 0.06 | 0.16 |
| X200082 | L-Lysine HCl | 65.05 | 68.31 | 36.62 | 49.73 | 27.37 | 67.50 | 68.74 | 105.35 |
| X200085 | 2i_L-Norleucine_D-Alloisoleucine | 12.93 | 11.58 | 7.29 | 8.13 | 2.62 | 13.74 | 3.22 | 20.06 |
| X200087 | 2i_Rutin _Quercetin-3-O-b-glucopyranosyl-7-O-a-rhamnopyranoside | 0.36 | 0.42 | 0.92 | 0.66 | 0.47 | 1.22 | 0.38 | 0.77 |
| X200089 | 2i_D-(+)-Melezitose monohydrate_1-Kestose | 0.71 | 0.53 | 0.81 | 0.27 | 0.12 | 0.23 | 0.04 | 0.54 |
| X260002 | gamma-Glu-PRENCSO | 0.77 | 1.61 | 0.99 | 0.85 | 0.10 | 0.39 | 0.36 | 0.53 |
| X260006 | MCSO | 12.59 | 33.57 | 8.23 | 13.04 | 11.02 | 1.68 | 5.37 | 4.93 |

| | | | | | | | | | |
|---|---|---|---|---|---|---|---|---|---|
| X260007 | Alliin | 24.48 | 44.98 | 21.42 | 36.37 | 13.21 | 30.51 | 17.57 | 25.60 |
| **ID** | **Annotation** | VNM_Mai Dinh | THA67-4 | THA16-5 | JPN_Taishu-san | SHA_Shanghi | JPN_Kikai-onodu | | |
| X00004 | 1-Amino-1-cyclopentanecarboxylic acid | 3.88 | 9.58 | 7.55 | 4.75 | 9.32 | 3.72 | | |
| X00010 | Citric acid,Anhydrous | 2.25 | 0.88 | 1.50 | 2.86 | 1.42 | 1.10 | | |
| X00039 | Adenine | 1.00 | 1.12 | 1.27 | 1.19 | 0.59 | 0.89 | | |
| X00049 | Glycine | 0.16 | 0.36 | 0.34 | 0.21 | 0.60 | 0.22 | | |
| X00054 | L-(-)-Phenylalanine | 0.80 | 1.02 | 1.27 | 1.51 | 2.02 | 2.33 | | |
| X00055 | L-Tryptophane | 0.97 | 1.03 | 1.99 | 2.79 | 1.37 | 2.44 | | |
| X00056 | L-Tyrosine | 0.28 | 0.59 | 0.84 | 0.89 | 0.48 | 0.88 | | |
| X00060 | Sucrose | 6.55 | 5.51 | 6.02 | 5.81 | 7.22 | 5.91 | | |
| X00095 | Methylmalonic acid | 0.29 | 0.35 | 0.31 | 0.24 | 0.24 | 0.32 | | |
| X00099 | Pyridoxamine dihydrochloride | 0.04 | 0.10 | 0.09 | 0.05 | 0.12 | 0.07 | | |
| X00187 | Cystathionine | 0.02 | 0.01 | 0.01 | 0.00 | 0.02 | 0.01 | | |
| X00188 | Cytidine,cell culture tested | 1.14 | 1.71 | 2.03 | 2.75 | 1.63 | 2.14 | | |
| X00212 | DL-2,3-Diaminopropionic acid monohydrochloride | 0.25 | 0.11 | 0.17 | 0.15 | 0.11 | 0.08 | | |
| X00218 | DL-threo-beta-Methylaspartic acid | 0.10 | 0.08 | 0.09 | 0.05 | 0.10 | 0.06 | | |
| X00241 | Guanosine | 0.28 | 0.27 | 0.29 | 0.60 | 0.47 | 0.27 | | |
| X00256 | Inosine | 0.22 | 0.17 | 0.21 | 0.20 | 0.34 | 0.20 | | |
| X00267 | L-Asparagine | 0.69 | 4.19 | 3.06 | 1.18 | 5.98 | 1.29 | | |
| X00268 | L-Aspartic acid | 4.33 | 4.52 | 4.82 | 2.73 | 7.87 | 2.62 | | |
| X00270 | L-Cystine | 0.10 | 0.04 | 0.11 | 0.05 | 0.06 | 0.04 | | |
| X00272 | L-Glutamic acid | 54.10 | 50.96 | 54.48 | 37.97 | 49.32 | 35.05 | | |
| X00273 | L-Glutamine | 10.80 | 38.67 | 27.02 | 17.54 | 46.48 | 15.50 | | |
| X00285 | L-Ornithine monohydrochloride | 0.15 | 0.85 | 0.64 | 0.29 | 1.18 | 0.46 | | |
| X00286 | L-Proline | 6.65 | 11.78 | 15.06 | 13.03 | 16.95 | 11.23 | | |
| X00287 | L-Pyroglutamic acid | 3.90 | 10.61 | 7.91 | 5.05 | 9.87 | 3.93 | | |
| X00288 | L-Serine | 6.15 | 9.94 | 6.15 | 6.58 | 14.76 | 4.92 | | |
| X00338 | S-Carboxymethyl-L-cysteine | 0.16 | 0.06 | 0.10 | 0.06 | 0.20 | 0.09 | | |
| X00344 | Succinic acid | 0.35 | 0.34 | 0.39 | 0.28 | 0.23 | 0.30 | | |
| X00364 | Tyramine | 0.50 | 0.92 | 0.74 | 0.68 | 1.12 | 0.76 | | |
| X00372 | Uridine-5'-monophosphate | 0.01 | 0.01 | 0.04 | 0.02 | 0.06 | 0.03 | | |
| X00379 | alpha-Lactose monohydrate | 0.67 | 0.44 | 0.55 | 0.47 | 0.65 | 0.53 | | |
| X00389 | gamma-Amino-n-butyric acid | 5.37 | 2.60 | 3.63 | 3.13 | 2.55 | 1.80 | | |
| X00392 | 6-Aminohexanoic acid | 0.70 | 0.73 | 1.21 | 1.21 | 0.88 | 1.15 | | |
| X00416 | Keracyanin Chloride | 1.69 | 3.21 | 3.72 | 2.26 | 2.02 | 1.39 | | |
| X00419 | Isorhamnetin-3-O-glucoside | 1.75 | 4.03 | 9.41 | 7.27 | 3.93 | 5.23 | | |
| X00422 | Kaempferol-3-O-glucoside | 10.58 | 20.55 | 23.24 | 21.48 | 12.59 | 20.79 | | |
| X00432 | Hyperoside | 0.72 | 2.51 | 6.10 | 4.89 | 2.59 | 3.60 | | |
| X00477 | O-Acetyl-L-serine hydrochloride | 0.50 | 0.91 | 0.51 | 0.41 | 0.50 | 0.59 | | |
| X00502 | L-Saccharopine | 0.19 | 0.31 | 0.44 | 0.32 | 0.21 | 0.26 | | |
| X00675 | 5'-Deoxy-5'-Methylthioadenosine | 1.94 | 1.71 | 1.29 | 1.61 | 2.49 | 1.43 | | |
| X00695 | L-allo-threonine | 2.18 | 2.48 | 2.67 | 2.28 | 3.23 | 2.36 | | |
| X00755 | 5-Aminovaleric acid | 2.96 | 2.50 | 3.47 | 2.95 | 3.86 | 2.96 | | |

| | | | | | | | | | |
|---|---|---|---|---|---|---|---|---|---|
| X00797 | L-Threonic acid hemicalciumsalt | 1.01 | 0.71 | 0.91 | 0.65 | 0.80 | 0.75 | | |
| X00849 | Hesperidin | 0.06 | 0.28 | 0.38 | 0.16 | 0.26 | 0.32 | | |
| X00853 | Luteolin-3',7-di-O-glucoside | 4.79 | 10.31 | 12.10 | 10.62 | 7.04 | 10.64 | | |
| X00854 | Luteolin-4'-O-glucoside | 23.39 | 46.78 | 56.18 | 48.22 | 29.13 | 47.02 | | |
| X00860 | Neohesperidin | 0.13 | 0.54 | 0.74 | 0.33 | 0.56 | 0.64 | | |
| X00865 | Poncirin | 0.04 | 0.13 | 0.14 | 0.08 | 0.10 | 0.08 | | |
| X00906 | Cysteinylglycine | 0.77 | 0.68 | 0.87 | 0.54 | 1.77 | 1.11 | | |
| X00907 | DL-Homocysteine | 0.02 | 0.02 | 0.01 | 0.01 | 0.02 | 0.02 | | |
| X00921 | Kaempferol-3-Rhamnoside-4"-Rhamnoside,-7-Rhamnoside | 0.27 | 0.15 | 0.16 | 0.11 | 0.20 | 0.28 | | |
| X00924 | Kaempferol-3-Glucuronide | 33.51 | 31.71 | 35.49 | 26.00 | 26.48 | 29.25 | | |
| X00933 | Quercetin-3-Glucuronide | 12.99 | 14.85 | 18.83 | 12.99 | 13.53 | 16.46 | | |
| X00943 | alpha-Methyl-DL-histidine dihydrochloride | 0.05 | 0.03 | 0.03 | 0.01 | 0.06 | 0.01 | | |
| X00971 | DL-5-Hydroxylysine hydrochloride | 0.02 | 0.01 | 0.01 | 0.02 | 0.01 | 0.02 | | |
| X00986 | L-Anserine | 0.03 | 0.05 | 0.06 | 0.07 | 0.04 | 0.07 | | |
| X01016 | Choline chloride | 45.14 | 37.44 | 34.69 | 35.60 | 31.12 | 33.71 | | |
| X01026 | Trigonelline hydrochloride | 1.87 | 1.55 | 2.42 | 2.84 | 2.54 | 2.43 | | |
| X01097 | DL-Pipecolinic acid | 2.63 | 6.58 | 5.52 | 3.28 | 6.40 | 2.58 | | |
| X01098 | Maleic acid | 0.12 | 0.07 | 0.06 | 0.07 | 0.03 | 0.06 | | |
| X01099 | D-(+)-Raffinose pentahydrate | 0.94 | 0.45 | 0.40 | 0.79 | 0.48 | 1.00 | | |
| X01144 | S-Methyl-L-cysteine | 0.70 | 0.72 | 0.87 | 0.72 | 0.50 | 0.65 | | |
| X01218 | Petunidin-3-O-beta-glucopyranoside | 3.69 | 2.71 | 3.33 | 5.89 | 2.53 | 5.10 | | |
| X01219 | Quercetin-3,4'-O-di-beta-glucopyranoside | 0.10 | 0.62 | 0.97 | 0.70 | 0.51 | 0.53 | | |
| X01220 | Cyanidin-3-O-(2"-O-beta-xylopyranosyl-beta-glucopyranoside) | 0.67 | 2.64 | 2.28 | 1.79 | 0.77 | 1.33 | | |
| X01223 | Delphinidin-3-O-(6"-O-alpha-rhamnopyranosyl-beta-glucopyranoside) | 0.97 | 1.98 | 3.25 | 1.81 | 1.65 | 2.03 | | |
| X01224 | Delphinidin-3-O-(2"-O-beta-xylopyranosyl-beta-glucopyranoside) | 0.18 | 0.21 | 0.46 | 0.47 | 0.22 | 0.38 | | |
| X01225 | Cyanidin-3-O-(2"-O-beta-xylopyranosyl-beta-glucopyranoside)-5-O-beta-glucopyranoside | 0.48 | 0.24 | 0.27 | 0.39 | 0.24 | 0.29 | | |
| X100024 | L-Leucine, (Cell Culture Reagent, Crystalline) | 0.73 | 1.02 | 1.56 | 1.69 | 1.09 | 1.77 | | |
| X100025 | L-Isoleucine | 0.61 | 0.70 | 1.26 | 1.12 | 0.80 | 1.21 | | |
| X100027 | Methionine sulfoxide | 0.20 | 0.33 | 0.49 | 0.42 | 0.30 | 0.33 | | |
| X100039 | Luteolin 7-O-glucuronide | 34.69 | 32.77 | 40.96 | 27.37 | 27.06 | 24.01 | | |
| X100043 | 2i_Leu_Ile | 6.50 | 8.85 | 14.26 | 14.29 | 9.62 | 14.64 | | |
| X100044 | 2i_Gln_Lys | 19.97 | 70.24 | 51.02 | 34.62 | 84.75 | 29.19 | | |

| | | | | | | | | | |
|---|---|---|---|---|---|---|---|---|---|
| X200004 | 3i_Isorhamnetin-3-O-rutinoside_Isorhamnetin-3-Glucoside-6"-Rhamnoside_Isorhamnetin-3-Galactoside-6"-Rhamnoside | 2.62 | 5.17 | 7.20 | 4.67 | 3.46 | 4.57 | | |
| X200007 | 2i_Cyanidin-3,5-di-O-glucoside chloride_Cyanidin-3-O-(2"-O-beta-glucopyranosyl-beta-glucopyranoside) | 5.52 | 10.58 | 12.36 | 12.30 | 7.72 | 11.56 | | |
| X200011 | 2i_Ideain chloride_Cyanidin-3-glucoside chloride | 20.52 | 47.29 | 53.51 | 47.14 | 27.64 | 46.43 | | |
| X200012 | 2i_Maritimein_luteolin-7-O-glucoside | 18.31 | 36.22 | 42.25 | 38.84 | 22.73 | 36.34 | | |
| X200017 | 2i_Syringetin-3-O-glucoside_Syringetin-3-O-galactoside | 0.01 | 0.97 | 1.94 | 1.36 | 0.94 | 1.05 | | |
| X200029 | 2i_alpha-D-Glucose-1-phosphate dipotassium salt dihydate_alpha-D-Galactose-1-phosphate dipotassium salt pentahydrate | 0.12 | 0.14 | 0.16 | 0.11 | 0.16 | 0.06 | | |
| X200030 | 2i_D-Mannose-6-phosphate barium salt hydrate_D-Mannose 6-phosphate mono sodium salt | 0.07 | 0.02 | 0.12 | 0.04 | 0.17 | 0.07 | | |
| X200031 | 4i_D-Fructose-6-phosphate disodium salt hydrate _D-Glucose-6-phosphate sodium salt _alpha-D-(+)-mannose-1-phosphate sodium salt hydrate_alpha-D(+)Mannose 1-phosphate bis(cyclohexylammonium) salt | 0.03 | 0.07 | 0.12 | 0.05 | 0.17 | 0.07 | | |
| X200034 | 2i_D(+)-Galactosamine hydrochloride_D-(+)-Glucosamine hydrochloride | 0.02 | 0.09 | 0.09 | 0.04 | 0.09 | 0.08 | | |
| X200042 | 2i_D-(+)-Cellobiose_Lactulose | 0.72 | 0.52 | 0.62 | 0.57 | 0.73 | 0.60 | | |
| X200043 | 6i_Melibiose hydrate_D-(+)-Turanose_Isomaltose_Gentiobiose_MelibiosePalatinose Monohydrate | 4.40 | 3.27 | 3.93 | 3.71 | 4.79 | 3.87 | | |
| X200047 | 4i_L(+)-Arginine HCl_N-alpha-Acetyl-L-ornithine_L-Citrulline | 1.36 | 4.38 | 6.28 | 4.83 | 8.02 | 6.66 | | |
| X200054 | DL-Cysteine | 0.02 | 0.01 | 0.01 | 0.01 | 0.03 | 0.01 | | |
| X200055 | 2i_Nicotinamide_Niacinamide | 0.51 | 0.70 | 0.63 | 0.66 | 0.59 | 0.50 | | |
| X200057 | 2i_L-Alanine_Sarcosine | 4.52 | 8.78 | 12.71 | 6.85 | 8.32 | 7.08 | | |
| X200060 | 2i_L-Norvaline_L-Valine | 4.63 | 4.17 | 5.37 | 4.44 | 6.63 | 4.73 | | |
| X200061 | 3i_L-Threonine_alpha-Methyl-DL-serine_L-Homoserine | 2.19 | 2.43 | 2.65 | 2.31 | 3.67 | 2.38 | | |
| X200063 | 3i_L-2-Aminobutyric acid_N,N-Dimethylglycine hydrochloride_N-Methyl-DL-Alanine | 8.81 | 7.43 | 6.88 | 7.42 | 6.69 | 6.88 | | |
| X200068 | DL-Malic acid | 3.82 | 2.16 | 2.17 | 1.75 | 1.71 | 1.91 | | |

| | | | | | | | | | |
|---|---|---|---|---|---|---|---|---|---|
| X200072 | L-CarnitineHCl | 0.01 | 0.03 | 0.06 | 0.01 | 0.02 | 0.04 | | |
| X200073 | DL-2-Aminoadipic Acid | 0.13 | 0.09 | 0.12 | 0.11 | 0.28 | 0.15 | | |
| X200082 | L-Lysine HCl | 20.02 | 70.29 | 51.19 | 33.82 | 84.42 | 29.60 | | |
| X200085 | 2i_L-Norleucine_D-Alloisoleucine | 6.43 | 8.69 | 12.84 | 14.02 | 8.97 | 14.91 | | |
| X200087 | 2i_Rutin _Quercetin-3-O-b-glucopyranosyl-7-O-a-rhamnopyranoside | 0.46 | 1.45 | 2.07 | 1.10 | 1.57 | 1.82 | | |
| X200089 | 2i_D-(+)-Melezitose monohydrate_1-Kestose | 0.81 | 0.51 | 0.37 | 0.80 | 0.43 | 0.92 | | |
| X260002 | gamma-Glu-PRENCSO | 1.05 | 0.83 | 0.68 | 0.77 | 1.18 | 1.04 | | |
| X260006 | MCSO | 6.62 | 0.41 | 0.40 | 1.19 | 4.97 | 1.27 | | |
| X260007 | Alliin | 27.88 | 13.53 | 21.06 | 14.96 | 48.93 | 21.65 | | |

**Supplementary Table S3.** Gradient condition for mass spectrometry analysis

| Time (min) | Flow (μL· min-1) | A (%) | B (%) |
|---|---|---|---|
| 0.25 | 0.24 | 99.9 | 0.1 |
| 0.4 | 0.24 | 91 | 9 |
| 0.8 | 0.24 | 83 | 17 |
| 1.9 | 0.24 | 0.1 | 99.9 |
| 2.1 | 0.24 | 0.1 | 99.9 |
| 2.11 | 0.24 | 99.9 | 0.1 |
| 2.7 | stope | | |

LC column: ACQUITY UPLC HSS T3 Column, 100 Å, 1.8 μm, 1 mm × 50 mm (Waters)

Colum oven temperature at 30℃

Mobile phase A: 0.1% v/v formic acid in water

Mobile phase B: 0.1% v/v formic acid in acetonitrile
